# Mössbauer Studies of Narrow Fractions of Fly Ash Formed after Combustion of Ekibastuz Coal

**DOI:** 10.3390/ma14237473

**Published:** 2021-12-06

**Authors:** Mikhail Vereshchak, Irina Manakova, Adilkhan Shokanov, Sayabek Sakhiyev

**Affiliations:** 1 Institute of Nuclear Physics, Ibragimov st. 1, Almaty 050032, Kazakhstan; mikhail.vereshchak@mail.ru; 2 Institute of Mathematics, Physics and Informatics, Abai Kazakh National Pedagogical University, Dostyk Av. 13, Almaty 050010, Kazakhstan; adilhan.shokanov@mail.ru; 3 Kazakh National Agrarian Research University, Abai Av. 8, Almaty 050010, Kazakhstan; ssayabek@yandex.ru

**Keywords:** Mössbauer spectroscopy, fly ash, granulometric and magnetic separation

## Abstract

Nuclear gamma-resonance spectroscopy on ^57^Fe nuclei, X-ray diffraction, and scanning electron microscopy have been used to study the narrow fractions of fly ash formed after combustion of the Ekibastuz coal. Two groups of samples of magnetic (ferrospheres) and non-magnetic type have been separated by granulometric and magnetic separation. A number of regularities associated with the granules size of fly ash have been established. According to the data of Mössbauer spectroscopy, a decrease in the magnetically ordered contribution has been identified with the growth of the particle size. After magnetic separation, iron in ferrospheres was found mainly in the structure of Fe_3_O_4_/γ-Fe_2_O_3_ and α-Fe_2_O_3_. The dominant phase was Fe_3_O_4_ (60–77%), the amount of which decreases with the growth of the grain size. With the growth of the particle size, the ratio of *[Fe]^tetra^/[Fe]^octa^* positions occupancy in Fe_3_O_4_ approaches 0.5; the structure of magnetite tends to the stoichiometric composition. α-Fe was found in the composition of ferrospheres, and a mechanism of its formation was proposed. The main components of the non-magnetic fractions of fly ash are mullite, hercynite, and silicate glass.

## 1. Introduction

Kazakhstan is a country with huge hydrocarbon reserves, and it occupies the 10th place in the world in terms of solid fuel reserves. The main source of fuel for thermal power plants (TPP) is the Ekibastuz coal basin, where coal is mined by the open method, which makes this type of solid fuel the cheapest, but it is characterized by a high ash content of ≈50% [1,2]. Therefore, a huge amount of the ash and slag mixture has been accumulated in the ash dumps of Kazakhstan, which creates a great threat to the environment [2,3,4]. It should be noted that with a sufficiently high level of the ash–slag waste production, only a small percentage of it is recycled. This leads to a rapid accumulation of waste from industrial coal combustion. In turn, it forces us to search for the ways of waste processing into valuable materials. The problems of ash–slag waste disposal in Russia, China, and Kazakhstan, where Ekibastuz coal is significant in the thermal power complex, were discussed in the works [3,4,5,6,7,8].

Currently, the ash–slag wastes are widely used in the construction industry [4,8,9] and road construction [10,11]. Ferrospheres and aluminosilicate microspheres in fly ashes are formed as a result of thermochemical and phase transformations during coal combustion [4,12,13,14]. Ferrospheres are the perspective materials for the development of catalysts for the reactions of oxidation of methane condensation and sorbents of liquid radioactive waste [12,13]. The most peculiar and valuable components of ash are cenospheres. They are hollow aluminosilicate balls of almost ideal shape with a smooth surface, from 10 to several hundred µm in diameter. Wall thickness is from 0.2 to 10 µm, T_melt_ = 1400–1500 °C, density is 580–690 kg·m^−3^ [15,16,17]. The unique properties of cenospheres have led to a wide range of their application in the production of lightweight heat-insulating materials, radio-transparent ceramics, lightweight mixtures, etc. There are a number of factors affecting the formation of microspheres with different microstructures. The most important of them are the types of boilers and combustion conditions. So, in the paper [6], significant differences were established in the phase composition of fly ashes as well as their components, depending on the heat–power characteristics of the boilers.

Many papers have been devoted to the study of fly ashes. It is shown in [4,6,7,10,13,18] that the main magnetic components of fly ashes are magnetite Fe_3_O_4_ and hematite α-Fe_2_O_3_. In addition, the presence of γ-Fe_2_O_3_ in fly ash was discovered in [6,7]. Fly ashes contain the paramagnetic components as mullite [19,20] and hercynite [6,20], as well as silicate glasses and various calcium silicates with the impurities of iron [7,20]. However, in contrast to [6,20], the presence of hercynite in fly ash was not found in [18,19]. Despite the fairly extensive research, the interpretation of the physical–chemical properties of fly ashes is still required. This work includes a detailed study of the narrow fractions of fly ashes subjected to separate granulometric and magnetic separation. Nuclear gamma-resonance spectroscopy (Mössbauer effect) is used as the main method of research. This method, equipped with a modern mathematical instrument, is the most informative (and in some cases the only one) for identifying the iron-containing phases. The Mössbauer core serves as probe in a solid state. It can be used to study the dynamic properties; structural, valence, and charge states of the Mössbauer atom; the phase composition; and the atomic, crystalline, magnetic, and electronic features of the structure of the studied substance [21,22,23,24,25,26,27,28]. Separation into magnetic and non-magnetic components makes it possible to facilitate the analysis of the chemical and phase state of iron in individual fractions of fly ashes, which is enriched by separation [15].

## 2. Materials and Methods

Fly ash from a pulverized combustion of coal in the Ekibastuz basin in the boiler unit furnace BKZ-420-140 KA2 (Barnaul Boiler Plant, Barnaul, Russia) of TPP-2 in Almaty at a flame temperature of 1500–1900 °C was used as an initial product to obtain the narrow fractions. The ash was collected on the electrostatic filter. The particle size ranged from 0.2 to 500 μm. From the analysis of the literature data [1,5,29], it follows that the average particle size of fly ash is usually 50–100 µm. Taking this fact into account, the separation of narrow fractions of microspheres was carried out by the standard sieves with the mesh size of 25, 40, and 100 μm. Four series of samples were prepared in this way. The results of granulometric separation are shown in Table 1. Furthermore, magnetic separation of each of the obtained fractions was completed in two stages. At the first stage, a barium oxide magnet (ferroalloy BaOFe_12_O_18_) was used, creating a field strength of 700 Oe. This enabled us to separate non-magnetic parts in a pure form from the fractions produced by granulometric separation. At the second stage, the remaining magnetic parts of the fractions were subjected to more thorough separation using a permanent magnet with a low field strength of ≈50 Oe. So, if usually ferrospheres in fly ash are from 3 to 17 wt % [4], then by dry and wet magnetic separation, the content of magnetic concentrate can be increased to 95 wt % or more [15]. This method enabled us to separate the ferrospheres with high iron content and estimate the amount of paramagnetic impurity, which is mechanically bound (apparently sintered) with the magnetic part of the separated samples. Thus, two groups of the narrow fraction samples have been prepared: magnetic and non-magnetic types.

The chemical state of iron in the crystal structure of fly ash narrow fractions was determined by Mössbauer spectroscopy (MS) at ^57^Fe nuclei. The spectra were recorded in transmission geometry on a MS-1104Em spectrometer (Research Institute of Southern Federal University, Rostov-on-Don, Russia) at room temperature. ^57^Co in a chromium matrix served as a source of γ-quanta. The Mössbauer spectra were processed using the software SpectrRelax (Version 2.4, Lomonosov Moscow State University, Moscow, Russia) [30]. X-ray diffraction (XRD) and scanning electron microscopy (SEM) were used as additional methods. The X-ray spectra were measured on a diffractometer Bruker D8 ADVANCE (Karlsruhe, Germany) with a Cu-K_α_ emitter in the Bragg–Brentano geometry. The card files ASTM and TCPDS were used to identify the crystalline phases. The morphology and elemental composition of fly ash narrow fractions was studied using a scanning electron microscope JEOL JSM-06610 (Tokyo, Japan) equipped with the IncaX-act energy dispersive analyzer with a resolution of 3 nm at accelerating voltage of 0.3–30 kV and accumulation time of 10 min. The elemental composition was recalculated for oxides; the amount was brought to 100%.

## 3. Results and Discussion

**Figure 1:**
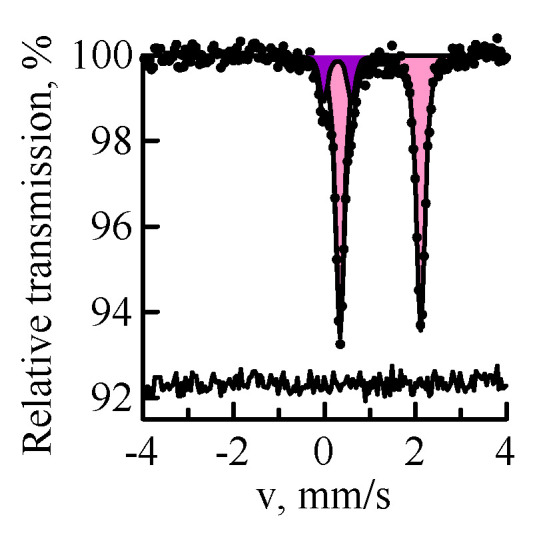
MS spectra of ^57^Fe nuclei of the Ekibastuz coal sample: siderite (pink); pyrite (purple).

Figure 1 shows the Mössbauer spectra of the sample of Ekibastuz coal. As can be seen, the main component of the coal spectrum is the doublet belonging to Fe^2+^ in the crystal structure of siderite FeCO_3_. In addition to siderite, coal contains Fe^3+^ in the structure of pyrite FeS_2_ (13 ± 1% of total spectrum). Thus, pyrite and siderite are the main precursors in the formation of ferrospheres during coal combustion. The mineral part of the Ekibastuz coal also contains clay minerals of the composition of kaolinite Al_2_Si_2_O_3_(OH)_4_, quartz α-SiO_2_, and carbonate CaCO_3_ [12]. In [31,32], magnetite was found as an impurity in coal, but the traces of this mineral were not found in this work.

**Figure 2:**
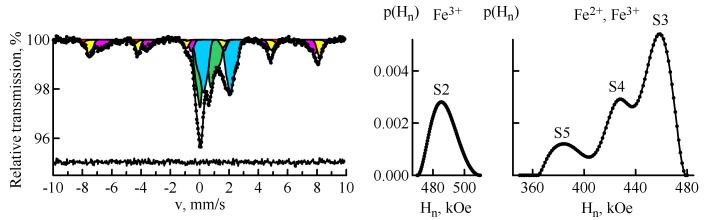
MS spectrum of ^57^Fe nuclei of the fly ash sample and restored distributions of hyperfine magnetic field *p*(*H_n_*): hematite (red); magnetite (yellow and magenta); the paramagnetic minerals (blue and green).

Figure 2 shows the Mössbauer spectrum of the sample of fly ash after the combustion of Ekibastuz coal and restored distributions of hyperfine magnetic field *p*(*H_n_*). As can be seen, the ash composition differs significantly from the original mineral components due to their significant changes under the influence of high temperatures. At the temperature of 585 °C, siderite, the content of which is ≈10% in the Ekibastuz coal [12], decomposes with the release of carbon dioxide CO_2_ and the formation of iron and its oxides, depending on the partial pressure of O_2_, CO, and CO_2_. Pyrite also decomposes with the subsequent oxidation of iron [6]. According to the data of chemical analysis, the main components of ash and slag are SiO (45–60%), Al_2_O_3_ (15–25%), Fe_2_O_3_ (5–15%), CaO (1.5–4.5%), and K_2_O (2.0–4.5%) [1].

As can be seen, the experimental Mössbauer spectrum of the fly ash is characterized by a rather complicated structure. The iron atoms occupy several positions in magnetically ordered and paramagnetic states. The spectrum cannot be described by a small discrete set of partial spectra. Therefore, for processing of the experimental data, distributions of hyperfine parameters were applied in the evaluation of the Mössbauer spectra [30]. The relative *Χ*^2^ was 1.17 ± 0.06.

Table 2 shows the Mössbauer parameters of the magnetic part of the MS spectrum of fly ash. The magnetic part of the spectrum consists of five sextets. The sextet *S1* is characterized by the parameters inherent to hematite α-Fe_2_O_3_. The sextet *S2* is defined by Fe^3+^ ions in the tetrahedral position of Fe_3_O_4_. The sextet *S3* corresponds to Fe^3+^ ions, and the sextets *S4–S5* correspond to Fe^2+^ ions in the octahedral position of Fe_3_O_4_. The presence of two types of sextets *S3* and *S4–S5* is associated with the significant non-stoichiometry of magnetite. As it is known, the ratio of tetrahedral position occupancy to octahedral position occupancy is *[Fe]^tetra^/[Fe]^octa^ = 0.5* for stoichiometric magnetite [33]. Electronic exchange is disturbed in case of non-stoichiometric magnetite Fe_3_O_4_. The sextet from Fe in the octahedral position decomposes into several non-equivalent states, and the Mössbauer spectrum gets a rather complex structure. In the literature, the spectrum of non-stoichiometric magnetite is often decomposed into three sextets: Fe^3+^ in the tetrahedral position, and Fe^3+^ and Fe^2+^ in the octahedral position. However, in practice, the picture is much more complicated, which is associated with the conditions of magnetite formation. The ratio *[Fe]^tetra^/[Fe]^octa^* is disturbed. The reasons for this disturbance are either isomorphic impurities in the octahedral position of Fe_3_O_4_, or the admixture of maghemite (and/or other impurities with close Mössbauer parameters) to magnetite. The isomorphic capacity of Fe_3_O_4_ is largely a function of temperature. At high temperatures, magnetite is able to capture the impurities of a number of elements [6]. In the present experiment, *[Fe]^tetra^/[Fe]^octa^* = 0.72.

The paramagnetic part of the fly ash spectrum was processed by two distributions of the quadrupole displacement, separately for Fe^2+^ and Fe^3+^ ions with the relative spectral areas 34.0 ± 0.9% and 30.4 ± 0.9%, respectively.

**Figure 3:**
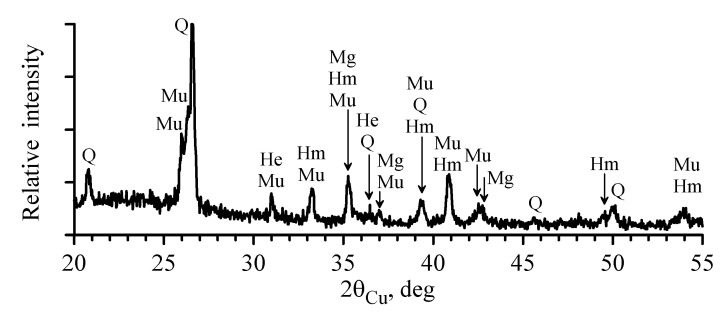
X-ray diffraction patterns of the fly ash sample.

Figure 3 shows the X-ray diffraction pattern of fly ash. The reflections of quartz SiO_2_ (Q), magnetite Fe_3_O_4_ (Mg), hematite α-Fe_2_O_3_ (Hm), mullite Al_6_Si_2_O_13_ (Mu), and hercynite FeAl_2_O_4_ (He) are observed against the background of a halo, indicating the presence of the amorphous phase.

**Figure 4:**
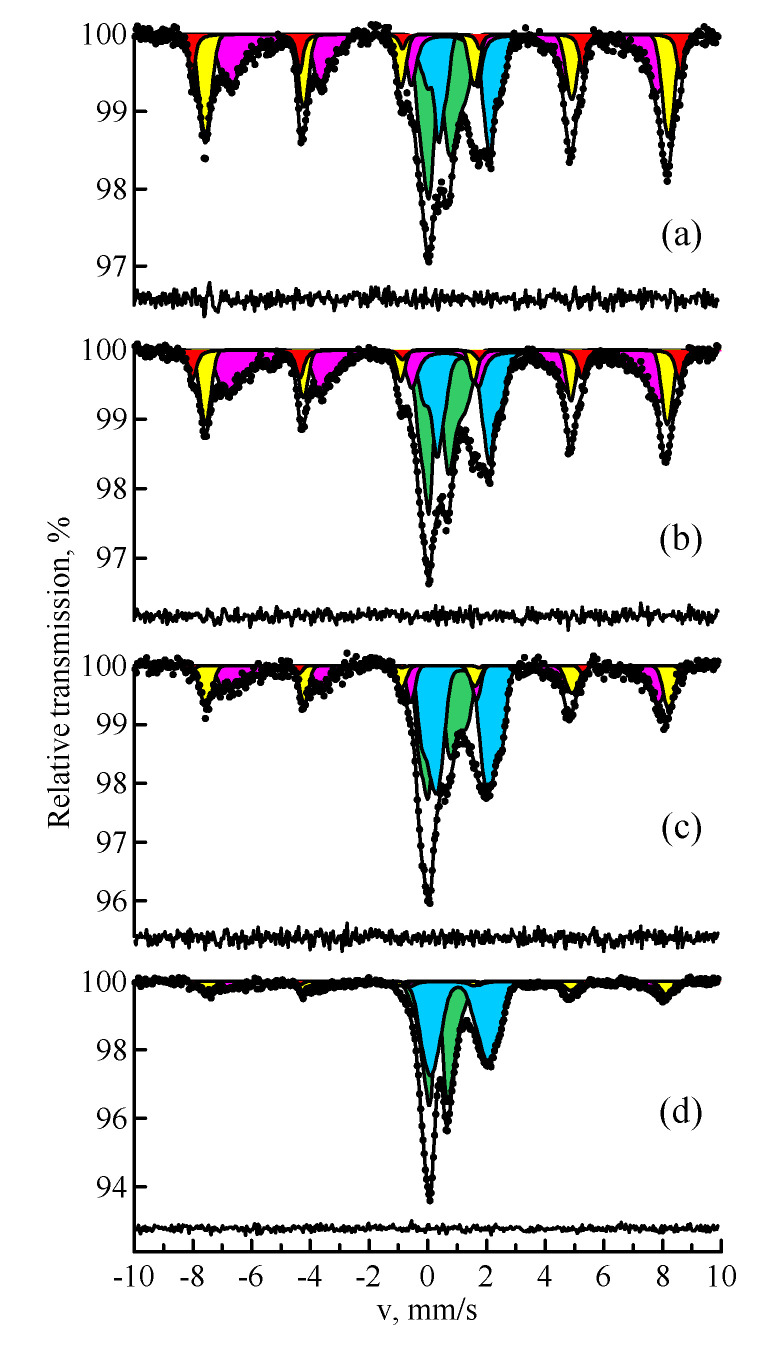
MS spectra of ^57^Fe nuclei of the samples of fly ash narrow fractions with a particle size of less than 25 µm (**a**), 25–40 µm (**b**), 40–100 µm (**c**), and more than 100 µm (**d**): hematite (red); magnetite (yellow and magenta); paramagnetic minerals (blue and green).

Figure 4 shows the Mössbauer spectra of the samples of fly ash narrow fractions produced after granulometric separation. It is easy to see that a decrease in the magnetic component is observed with the growth of the granule size. In a sample with a particle size of more than 100 μm, the content of ferroxides is minimal; the paramagnetic components are predominant. The distribution of iron over magnetically ordered and paramagnetic phases in the Mössbauer spectra of fly ash narrow fractions is presented in Table 3.

**Figure 5:**
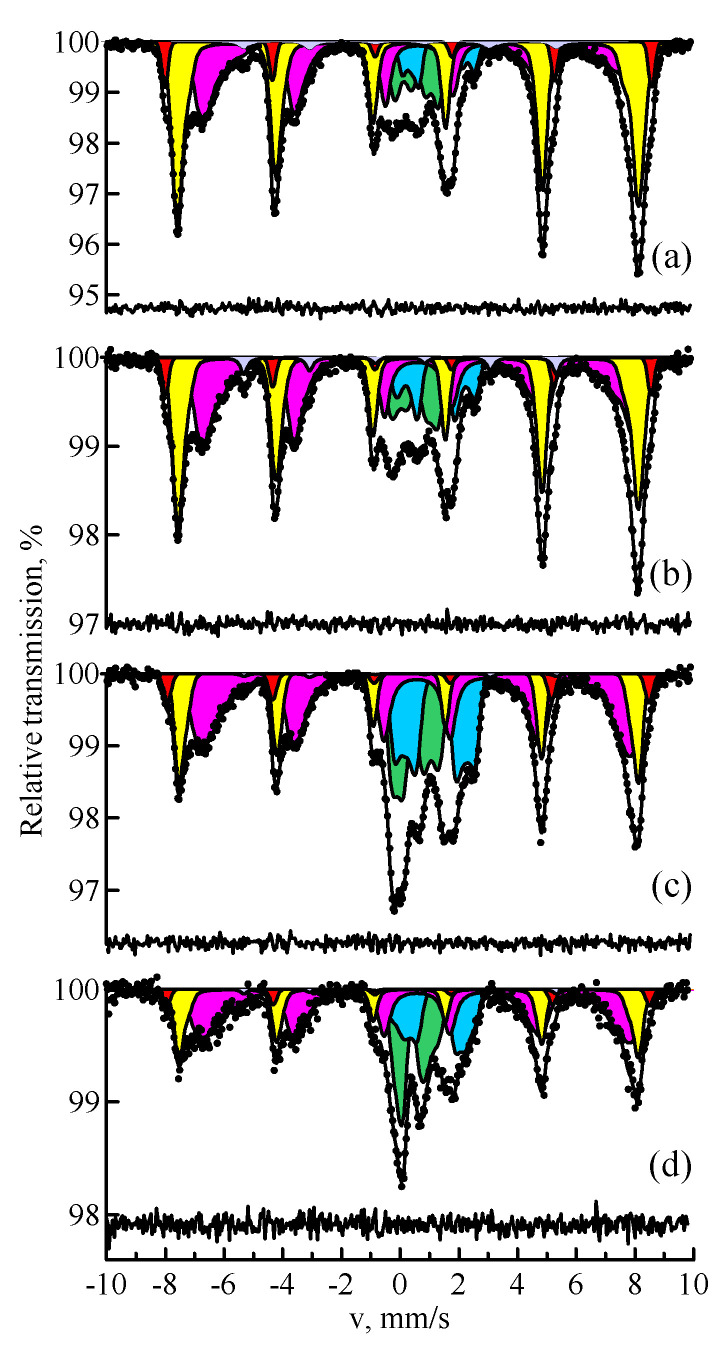
MS spectra of ^57^Fe nuclei of the samples of narrow magnetic fractions of fly ash with a particle size of less than 25 µm (**a**), 25–40 µm (**b**), 40–100 µm (**c**), and more than 100 µm (**d**): hematite (red); magnetite (yellow and magenta); the paramagnetic minerals (blue and green).

To study ferrospheres and aluminosilicate microspheres, the fractions separated from fly ash by the granulometric method were subjected to magnetic separation. Figure 5 shows the Mössbauer spectra of the samples of narrow magnetic fractions that resulted from the second stage of magnetic separation. Table 4 shows the Mössbauer parameters of the magnetic phases for the spectrum of the sample with particles less than 25 μm. Table 5 shows the distribution of iron over magnetically ordered phases versus particle size. It can be seen that iron in ferrospheres is found mainly in the structure of Fe_3_O_4_/γ-Fe_2_O_3_ and α-Fe_2_O_3_. Moreover, the dominant phase is Fe_3_O_4_ (60–77%), the amount of which decreases with the growth of the granule size. This is associated with the fact that Fe_3_O_4_ is formed from α-Fe_2_O_3_ at temperatures above 1400 °C. Probably, the equimolar oxidation of Fe_3_O_4_ to γ-Fe_2_O_3_ occurs upon cooling from high temperatures. Moreover, this process occurs in smaller granules that have greater contact with the oxidizing atmosphere in the heating unit.

In addition to the magnetically ordered state, the samples contain Fe^2+^ and Fe^3+^ ions in a paramagnetic structure. In accordance with [4,7,12], mullite crystals are precipitated on the surface of shells and inside the globules of magnetic microspheres with a high aluminum content. It follows from [6] that the paramagnetic part of the spectrum of magnetic microspheres also contains mullite. In addition, there is hercynite and aluminosilicate glass with the admixture of iron.

Natural metallic iron was not detected in the Mössbauer spectrum of the fly ash sample before magnetic separation. Apparently, this is caused by the very low content of α-Fe for its identification in such a complex spectrum of fly ash. This became possible in the spectra of the separated magnetic samples. In the spectra of the samples of fly ash narrow fractions, in addition to α-Fe_2_O_3_ and Fe_3_O_4_, there is a small amount of α-Fe. Iron reduction is in principle possible when the particles pass through a flare zone with a reducing atmosphere. In the combustion unit, both oxidizing and reducing atmosphere are formed, depending on the amount of oxide and carbon dioxide in the gas mixture. If the CO_2_/CO ratio is greater than a certain value, then iron will be reduced to metal, which is followed by encapsulation into aluminosilicate or ferrosphere globules.

One more interesting experimental fact draws attention. Table 6 shows the ratio of *[Fe]^tetra^/[Fe]^octa^* positions occupancy in Fe_3_O_4_ in the spectra of the narrow magnetic fraction samples. It can be seen that with the growth of particle size above 40 μm, the ratio *[Fe]^tetra^/[Fe]^octa^* decreases; the structure of magnetite tends to the stoichiometric composition, which can be explained by the fact that fine particles can contain Fe_3_O_4_/γ-Fe_2_O_3_ and isomorphic impurities in the octahedral position of magnetite. Small particles will have a higher oxidation rate due to greater contact with the gaseous oxidizing atmosphere, which is consistent with [7].

In addition to the magnetically ordered phases, the narrow magnetic fractions of fly ash contain a paramagnetic impurity mechanically bound to the magnetic part, and its content increases with the growth of the particle size.

**Figure 6:**
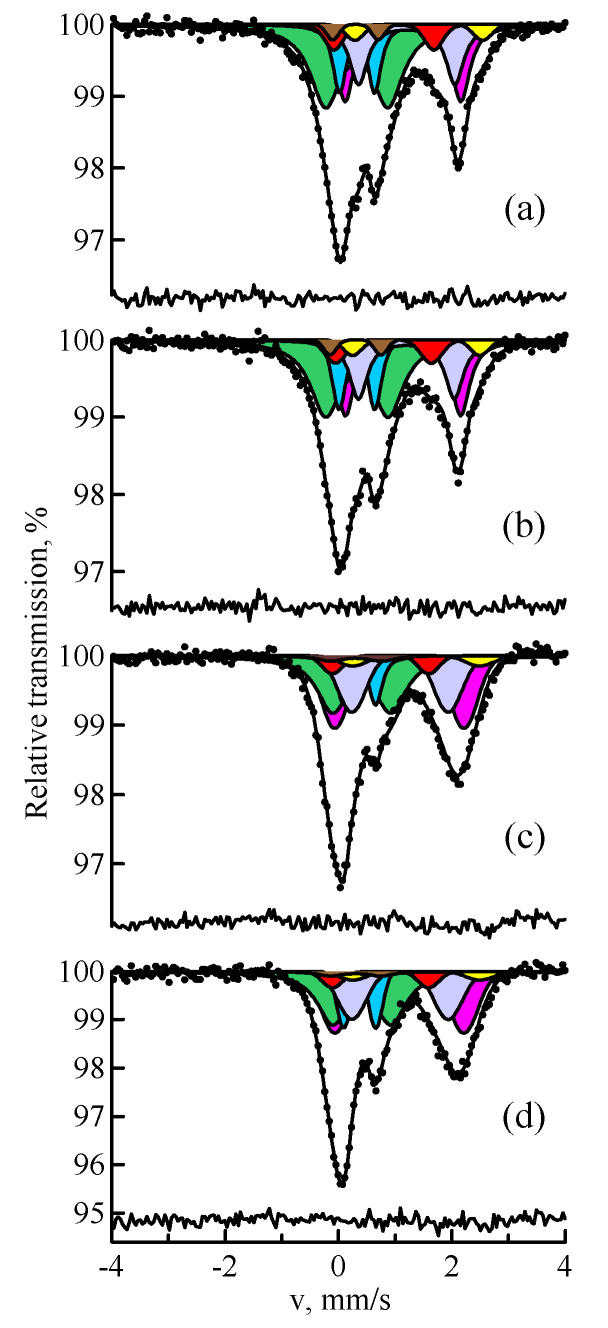
MS spectra of ^57^Fe nuclei of the samples of narrow non-magnetic fractions of fly ash with a particle size of less than 25 µm (**a**), 25–40 µm (**b**), 40–100 µm (**c**), and more than 100 µm (**d**): mullite (green, blue, and red); hercynite (powder blue, yellow, and brown); silicate (magenta).

Figure 6 shows the Mössbauer spectra of the samples of narrow non-magnetic fractions. Taking into account the similarity of the spectra of the samples with the particles of different sizes, Table 7 shows the Mössbauer parameters only for the spectrum of the sample with the particles less than 25 μm. The MS spectrum was processed by model decoding using seven doublets. *D1–D3* doublets are caused by Fe^2+^ ions in the tetrahedral environment and Fe^2+^, Fe^3+^ ions in the octahedral environment in the hercynite crystal lattice. The *D4–D6* doublets characterize Fe^3+^ ions in the octahedral environment and Fe^3+^, Fe^2+^ ions in the tetrahedral environment in the mullite crystal lattice. *D7* doublet refers to Fe^2+^ in the octahedral environment of silicate glass [6,19]. Table 8 shows the phase composition of the narrow non-magnetic fractions.

The paramagnetic impurity mechanically bound to the magnetic part of the separated samples (see Figure 5) is identical in quality to the non-magnetic fraction of fly ash, the main components of which are mullite, hercynite, and silicate glass.

**Figure 7:**
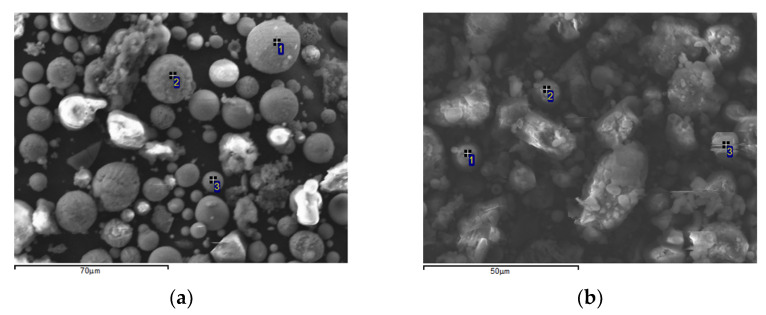
SEM images of magnetic (**a**) and non-magnetic (**b**) narrow fractions of fly ash with a particle size less than 25 μm.

Figure 7 shows the SEM images of magnetic (a) and non-magnetic (b) fly ash fractions with particle size less than 25 μm. Table 9 and Table 10 show the elemental composition of the particles marked on the SEM images. Figure 7a shows a cluster of nearly perfect spherical globules of various sizes. As the particle size decreases, their spheroidality improves. There are globules with both smooth and embossed surfaces. The iron content in them varies from 37.43 to 85.26 wt %, the content of aluminum is from 1.76 to 14 wt %, and the content of silicon is from 3.52 to 22.53 wt %. It can be seen from Table 6 that in the magnetic narrow fractions of fly ash with the granule size less than 25 μm, the ratio of positions occupancy *[Fe]^tetra^/[Fe]^octa^* = 1.16 ± 0.08. Such a significant violation of stoichiometry in magnetite cannot be provided by isomorphic impurities. The content of magnesium and calcium in the magnetic fractions with granule size less than 25 μm is higher than in the magnetic fractions of granules of larger sizes. In addition, the content of magnesium in the magnetic fraction is significantly higher than in the non-magnetic fraction. Apparently, these elements are precursors in the formation of magnesium ferrites, which can contribute to the Mössbauer spectrum of the magnetic fraction of the finely dispersed granules of fly ash. Particles of the non-magnetic fraction are mostly of indefinite shape (Figure 7b), silicon is the predominant element in them—from 53.84 to 66.67 wt %.

Thus, the study of granular narrow fractions by the SEM method made it possible to confirm, and in some cases to clarify, the conclusions made according to the results of Mössbauer studies.

## 4. Conclusions

The studies of the narrow fractions of fly ash resulted from combustion of Ekibastuz coal have been completed. Two groups of samples of the magnetic and non-magnetic type with the grain size less than 25 μm, 25–40 μm, 40–100 μm, and more than 100 μm were identified by granulometric and magnetic separation. Several regularities have been revealed related to the size of granules and the phase composition of the narrow fractions of fly ash.

According to MS data, after granulometric separation, a decrease in the magnetically ordered contribution was established with the growth of the particle size. In the sample with a particle size of more than 100 μm, the content of ferroxides is minimal; the minerals mullite and hercynite are predominant. Labeled magnetic particles less than 25 µm in size can be used as indicators in those fields of ferrospheres application, where materials with strong magnetic properties are required.

After magnetic separation, iron in ferrospheres is found mainly in the structure of Fe_3_O_4_/γ-Fe_2_O_3_ and α-Fe_2_O_3_. Moreover, the dominant phase is Fe_3_O_4_ (60–77%), the amount of which decreases with the growth of the granules size. It was found that with the growth of the particle size, the ratio of the positions occupancy *[Fe]^tetra^/[Fe]^octa^* in Fe_3_O_4_ approaches 0.5; the structure of magnetite tends to the stoichiometric composition. This is caused by the fact that Fe_3_O_4_ is formed from α-Fe_2_O_3_ at the temperatures above 1400 °C. Probably, equimolar oxidation of Fe_3_O_4_ to γ-Fe_2_O_3_ occurs upon cooling from high temperatures. Moreover, this process occurs in smaller granules that have greater contact with the oxidizing atmosphere in the heating unit. α-Fe was found in the composition of ferrospheres, and a mechanism of its formation was proposed. The paramagnetic impurity, which is mechanically bound to the magnetic part of the separated samples by its qualitative composition, is identical to the non-magnetic fraction of fly ash, the main components of which are mullite, hercynite, and silicate glass. The content of paramagnetic impurity in the magnetic fraction increases with the growth of the particle size.

## Figures and Tables

**Table 1:** Content of narrow fractions in fly ash after granulometric separation.

Particle Size, µm	Content, wt %
<25	7.60
25 ÷ 40	17.27
40 ÷ 100	36.80
>100	38.33

**Table 2:** Hyperfine parameters of the magnetic phases obtained from the MS spectrum of fly ash.

Subspectrum	*I*, %	*δ*, mm/s	*2ε*, mm/s	*H_n_*, kOe	Assignment
*S1*	3.3 ± 0.9	0.39 ± 0.02	−0.09 ± 0.04	516 ± 1	Hematite
*S2*	13.5 ± 0.9	0.33 ± 0.01	0.01 ± 0.01	485 ± 1	Magnetite *[Fe^3+^]^tetra^*
*S3*	11.0 ± 0.7	0.53 ± 0.01	−0.04 ± 0.01	459 ± 2	Magnetite *[Fe^3+^]^octa^*
*S4*	5.4 ± 0.4	0.58 ± 0.01	0.09 ± 0.01	429 ± 2	Magnetite *[Fe^2+^]^octa^*
*S5*	2.4 ± 0.3	0.66 ± 0.02	0.28 ± 0.04	384 ± 2	Magnetite *[Fe^2+^]^octa^*

*I*: relative spectral area; *δ*: isomer shift relative to metallic iron at 300 K; *ε*: quadrupole shift; *H_n_*: hyperfine field at ^57^Fe nuclei at maximum of the distribution.

**Table 3:** Distribution of iron over magnetically ordered (*I_m_*) and paramagnetic (*I_pm_*) phases in narrow fractions of fly ash.

Particle Size, µm	*I_m_*, %	*I_pm_*, %
<25	62 ± 4	38 ± 2
25 ÷ 40	55 ± 3	45 ± 1
40 ÷ 100	37 ± 2	63 ± 1
>100	19 ± 1	81 ± 1

**Table 4:** Hyperfine parameters of the magnetic phases obtained from the MS spectrum of the sample with the particles less than 25 μm.

Subspectrum	*I*, %	*δ*, mm/s	*2ε*, mm/s	*H_n_*, kOe	Assignment
*S1*	4.9 ± 0.8	0.37 ± 0.01	−0.09 ± 0.01	514 ± 2	Hematite
*S2*	41.5 ± 0.9	0.29 ± 0.01	−0.04 ± 0.01	487 ± 2	Magnetite *[Fe^3+^]^tetra^*
*S3*	14.2 ± 0.9	0.68 ± 0.01	0.01 ± 0.01	463 ± 2	Magnetite *[Fe^3+^]^octa^*
*S4*	15.1 ± 0.9	0.65 ± 0.01	0.02 ± 0.01	436 ± 3	Magnetite *[Fe^2+^]^octa^*
*S5*	6.4 ± 0.7	0.59 ± 0.02	0.05 ± 0.01	399 ± 3	Magnetite *[Fe^2+^]^octa^*
*S6*	2.1 ± 0.6	0.00 ± 0.03	0.09 ± 0.01	329 ± 2	α-Fe

**Table 5:** Distribution of iron over magnetically ordered phases in the magnetic narrow fractions of fly ash.

Particle Size, µm	α-Fe, %	α-Fe_2_O_3_, %	Fe_3_O_4_/γ-Fe_2_O_3_, %
<25	2 ± 1	5 ± 1	77 ± 3
25–40	3 ± 1	4 ± 1	74 ± 3
40–100	1 ± 1	4 ± 1	60 ± 2
>100	1 ± 1	3 ± 1	61 ± 5

**Table 6:** The ratio of the positions occupancy in Fe_3_O_4_.

Particle Size, µm	*[Fe]^tetra^/[Fe]^octa^*
<25	1.16 ± 0.08
25–40	0.88 ± 0.06
40–100	0.46 ± 0.04
>100	0.47 ± 0.09

**Table 7:** Hyperfine parameters of the non-magnetic fraction obtained from the MS spectrum of the sample with the particles less than 25 μm.

Subspectrum	*I*, %	*δ*, mm/s	Δ, mm/s	Assignment
*D1*	17.0 ± 0.3	1.20 ± 0.03	1.70 ± 0.06	Hercynite *[Fe^2+^]^tetra^*
*D2*	3.1 ± 0.1	1.41 ± 0.09	2.26 ± 0.09	Hercynite *[Fe^2+^]^octa^*
*D3*	2.0 ± 0.1	0.30 ± 0.09	0.80 ± 0.09	Hercynite *[Fe^3+^]^octa^*
*D4*	37.3 ± 0.9	0.32 ± 0.02	1.14 ± 0.06	Mullite *[Fe^3+^]^octa^*
*D5*	13.1 ± 0.2	0.33 ± 0.03	0.62 ± 0.04	Mullite *[Fe^3+^]^tetra^*
*D6*	5.6 ± 0.1	0.80 ± 0.09	1.76 ± 0.09	Mullite *[Fe^2+^]^tetra^*
*D7*	21.9 ± 0.4	1.14 ± 0.02	2.04 ± 0.02	Silicate *[Fe^2+^]^octa^*

**Δ**: quadrupole splitting.

**Table 8:** Phase composition (%) of narrow non-magnetic fractions.

Particle Size, µm	Hercynite	Mullite	Silicates
<25	22 ± 1	56 ± 1	22 ± 1
25–40	23 ± 1	54 ± 1	23 ± 1
40–100	27 ± 1	47 ± 2	26 ± 1
>100	26 ± 1	49 ± 1	25 ± 1

**Table 9:** Elemental composition (wt %) of narrow magnetic fraction of fly ash with a particle size less than 25 μm.

Particle	MgO	Al_2_O_3_	SiO_2_	P_2_O_5_	SO_3_	CaO	TiO_2_	MnO	Fe_2_O_3_
1	5.01	1.76	3.52	0.67	0.80	1.06	0.00	1.92	85.26
2	3.08	3.35	19.68	1.67	0.12	2.48	0.26	1.80	67.55
3	5.82	14.00	22.53	5.83	0.00	13.21	0.20	0.99	37.43

**Table 10:** Elemental composition (wt %) of narrow non-magnetic fraction of fly ash with a particle size less than 25 μm.

Particle	Na_2_O	MgO	Al_2_O_3_	SiO_2_	P_2_O_5_	SO_3_	K_2_O	CaO	TiO_2_	MnO	Fe_2_O_3_	Cu_2_O	ZnO
1	0.96	1.58	13.47	61.65		1.08	0.83	4.68	0.29	0.29	11.71	0.41	0.49
2	0.65	0.82	19.99	66.67	0.28	0.26	0.86	2.68	0.67	0.42	6.29	0.33	
3	0.74	0.58	28.41	53.84	0.94		0.79	1.62	1.22	0.31	11.25	0.25	

## Data Availability

The data presented in this study are available on request from the corresponding author.
